# Retrospective MRI analysis of 418 adult shoulder joints to assess the physiological morphology of the glenoid in a low-grade osteoarthritic population

**DOI:** 10.1186/s12880-025-01568-6

**Published:** 2025-01-31

**Authors:** Marc-Pascal Meier, Lars Erik Brandt, Mark-Tilmann Seitz, Paul Jonathan Roch, Katharina Jäckle, Ali Seif Amir Hosseini, Wolfgang Lehmann, Thelonius Hawellek

**Affiliations:** 1 Department of Trauma Surgery, Orthopaedics and Plastic Surgery, University Medical Center Goettingen, Robert-Koch-Straße 40, 37075 Göttingen, Germany; 2 Department of Clinical and Interventional Radiology, University Medical Center Goettingen, Robert-Koch-Straße 40, 37075 Göttingen, Germany

**Keywords:** Glenoidal morphology, Glenoidal reference, MRI, Shoulder morphology, Shoulder arthroplasty

## Abstract

**Background:**

Due to the difference in size between the humeral head and the glenoid, the shoulder joint is prone to instability. Therefore, the reconstruction of the physiological joint morphology is of great importance in shoulder joint preservation and replacement surgery. The aim of this study was to describe physiological reference parameters for the morphology of the glenoid for the first time.

**Material and methods:**

MRI images of the shoulder joints of 418 patients (mean age: 50.6 years [± 16.3]) were retrospectively analysed in a low-grade osteoarthritic population. The glenoid distance in coronal (GDc) and axial view (GDa), glenoid inclination (GI) and version (GV) as well as scapula neck length (SNL) were measured. Parameters were studied in association for age, gender, side and degeneration grade.

**Results:**

Mean GDc was 33.4 mm (± 3.6), mean GDa 26.8 mm (± 3.2), mean GI 10.5° (± 6.4), mean GV -0.4 mm (± 5.4) and mean SNL was 33.4 mm (± 4.7). GDa was significant higher in right shoulders (*p* < 0.001). GDc and GDa showed significant higher mean values in older patients (*p* < 0.001) and in shoulders with more severe degenerative changes (*p* < 0.05). While GDc, GDa and SNL were significant larger in male patients (*p* < 0.001), GI had a higher mean value in female shoulders (*p* = 0.021).

**Conclusion:**

Age, gender and shoulder joint degeneration influence changes in the morphological parameters of the glenoid. These findings have to be considered in shoulder diagnostics and surgery.

**Clinical trial number:**

Not applicable.

## Highlights

418 shoulder MRIs were retrospectively evaluated for glenoidal distance in coronal and axial view, glenoidal inclination, glenoidal version as well as scapula neck length.

Significant side-, age- and gender-specific differences were detected.

The analysis of the glenoidal version showed a slight retroversion position on average.

The glenoidal distance parameters appear to increase as part of the ageing process due to degenerative changes.

These results should be considered in shoulder diagnostics and surgery.

## Introduction

The morphology of the shoulder joint is highly complex [1–3]. In its function as a spherical joint, it has three degrees of freedom [4, 5]. A morphological peculiarity is the unequal size ratio of the articulation partners. Due to the joint pairing between the significantly larger humeral head and the smaller glenoid, the shoulder joint has a high range of motion, but at the same time is susceptible to instability in the event of suboptimal joint configuration [4–8]. Instability can be an expression of a pathological change in the morphology of the shoulder joint, as well as causing long-term degenerative damage [9–12]. In particular, the morphological constitution of the glenoid is crucial for joint stability [13]. There is a prevailing agreement in the current literature that chronic shoulder instability in young patients should be treated surgically, as the risk of premature osteoarthritis development is otherwise significantly increased [12]. Special attention should be paid to the glenoid in surgical treatment, as Vetter et al. were able to show that degenerative changes in the humeral head correlate with the morphology of the glenoid [14].

Furthermore, the positioning of the glenoid component is essential for joint stability after arthroplasty of the shoulder [15, 16]. Whether implanting the anatomical total shoulder joint arthroplasty or the reverse type, malpositioning of the glenoid component leads to instability and in the worst case to dislocation [16–18].

In this context, the three-dimensional morphology of the glenoid is of great importance in therapy of shoulder joint instability. In order to achieve an optimal postoperative result in case of glenoid fractures in combination with labral lesion or heavy joint degeneration, the best possible reconstruction of the physiological glenoid morphology is required for both joint-preserving and joint-replacing therapy [19, 20]. However, this requires valid ranges of physiological reference values for preoperative diagnostics and planning, especially in severe defect situations. Indeed, there are only a few studies in the current literature that examine the morphology of the glenoid in a radiologically low-level osteoarthritic population [21, 22]. In addition, some of the published references are based on conventional radiographs [22, 23]. There is a consensus that cross-sectional imaging is required for optimal radiological assessment of shoulder morphology [23–26].

The aim of the present study was therefore to define physiological reference values for glenoid morphology using cross-sectional imaging in a low-grade osteoarthritic population in order to optimize diagnostics and surgical therapy of shoulder joint instability in the native and arthroplasty joint. In addition, side-, age-, gender-, and degeneration-specific differences in glenoid morphology should be analysed.

## Materials and methods

### Patients

Between 2013 and 2021, a total of *n* = 2,629 patients underwent a magnetic resonance imaging of the shoulder joint in the Department for Diagnostic and Interventional Radiology of University Medical Center Goettingen. These MRIs were reviewed retrospectively. The aim of the analysis was to examine the physiological morphology of the glenoid in a low-grade osteoarthritic population. After applying inclusion and exclusion criteria, 418 patients were included in the final analysis. The study collective was divided into two age groups (20–50 and > 50 years). The study was approved by the local ethics committee (IRB number 17/5/22) and performed in accordance with the principles expressed in the Declaration of Helsinki. Without exception, the evaluated MRI were taken as part of routine diagnostics because of clinical symptoms. All MRIs were assessed by a senior radiologist and LEB, MPM, ASAH and TH to exclude extended structural injuries or heavy joint degeneration.

### Inclusion criteria

All examinations accessible via PACS system (Picture Archiving and Communication System) between 01.01.2013 to 31.12.2021 were initially included in the study. Out of these, all patients with an age of 20 years or more were included. All MRI scans were performed on patients to assess shoulder joint pathologies. Only patients with a Kellgren/Lawrence score [27] < 3 were classified as “healthy shoulder joint” (SJH). Patients with Kellgren/Lawrence Score of ≥ 3 were classified as “degenerative shoulder joint” (SJD). All MRIs were examined by the internal radiology department as part of the clinical diagnostic procedure. Every report was re-evaluated by LEB, MPM, ASAH and TH in a blinded fashion.

### Exclusion criteria

**Fig. 1:**
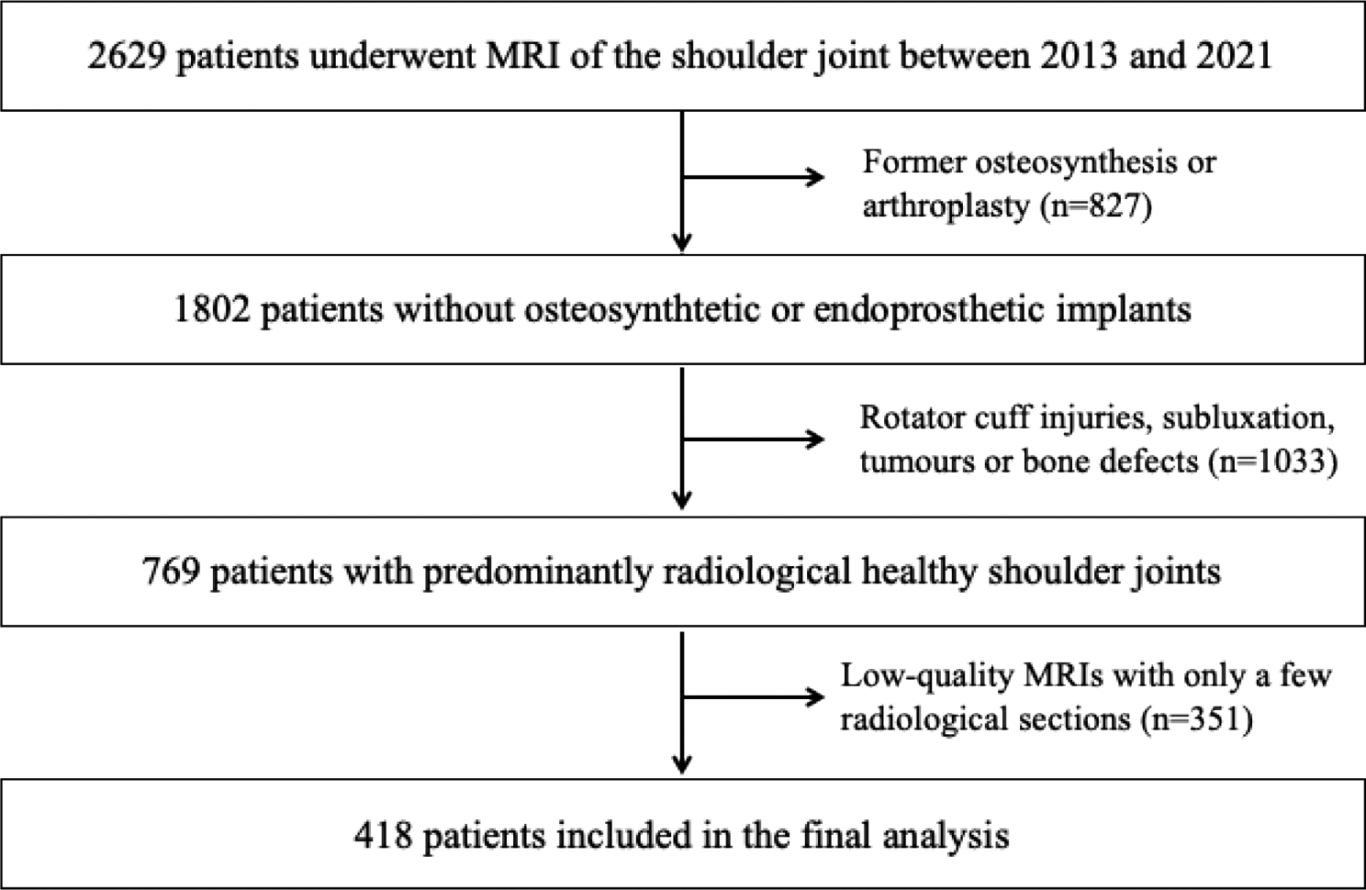
Flowchart of inclusion and exclusion process of the initial collective: 2629 patients who underwent MRI of the shoulder joint between 2013 and 2021 were examined. Patients with former osteosynthesis or arthroplasty (*n* = 827), detected injuries, tumours or bone defects (*n* = 1033), as well as MRIs with insufficient quality standard (*n* = 351) were excluded. In consequence, 418 patients were included in the final analysis

All patients with fractures, osteonecrosis, dysplasia or tumours were excluded. Patients who had undergone osteosynthesis or arthroplasty were likewise excluded. Similarly, the data did not include patients who had any other implants after shoulder joint preservation surgery. Patients with humeral head suluxation and extensive injuries of the rotator cuff were excluded, too. Low-quality MRIs (based on only a few radiological sections), were excluded. In addition, all MRIs with imaging artefacts were ruled out. Figure 1 shows a flowchart of the inclusion and exclusion procedure of the initial study collective.

### MRI analysis, parameters and methods of measurement

The “Magnetom Vida” from “Siemens Healthineers” was used as the standardized MRI scanner. MRI examinations of the shoulder were performed using a standard imaging protocol that adhered to the guidelines recommended by the European Society of Skeletal Radiology (ESSR). The scans were conducted with the patient in a supine position, with the arm in a neutral position to ensure optimal imaging of the shoulder joint structures.The imaging protocol included the following sequences:

T1-weighted Spin Echo (SE) in the coronal oblique plane with a slice thickness of 3 mm, used for anatomical evaluation and fat-containing structures.

Proton Density-weighted (PD) fat-Saturated images in the coronal oblique, sagittal oblique, and axial planes, with a slice thickness of 3 mm, to assess soft tissue and cartilage abnormalities while suppressing fat signal. The slice thickness across all sequences was maintained at 3 mm with an interslice gap of 0.3–0.5 mm to ensure high-resolution imaging while minimizing artifact overlap. All imaging was performed with attention to standardized field-of-view (FOV) and matrix size parameters as recommended by the ESSR guidelines to ensure consistency and diagnostic accuracy.

**Fig. 2:**
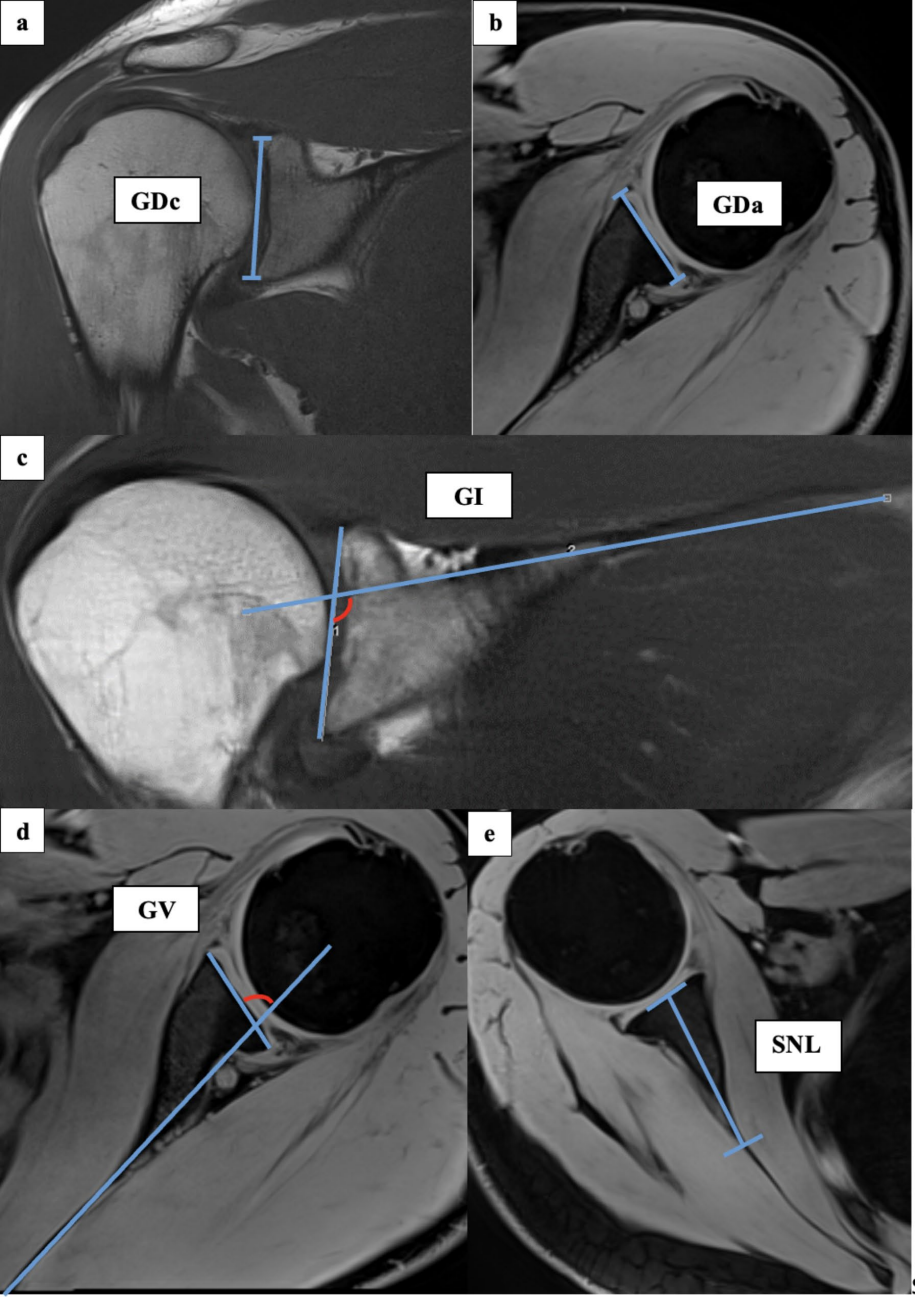
Exemplary depiction of the measurements of the glenoidal distance in coronal (GDc, **a**) and axial view (GDa, **b**), glenoidal inclination (GI, **c**), glenoidal version (GV, **d**) and scapula neck length (SNL, **e**): The measurements of GDc, GI and SNL were all performed in coronal view of the shoulder joint in MRIs. GDa and GV was measured in the axial view. To determine GD, the length of the maximum extension of the glenoid articular surface was measured in both the coronal (GDc, **a**) and axial (Goa, **b**) dimensions. In order to calculate the GI, a tangent was first applied to the cranial and caudal boundaries of the glenoid in the coronal plane. A linear line was then placed through the superior border of the scapula. Of the angles formed at the intersection of the two lines, the inferior medial angle was used for further calculation. From this angle, 90° were subtracted, resulting in GI (**c**). The same principle was used to determine the tangent and corresponding straight line in the axial sectional plane. For the further calculation the ventral lateral angle at the crossing point was used. From this angle, 90° were subtracted, resulting in GV (**d**). To determine SNL (**e**), the widest extent from the joint surface side to the cortical end of the medial scapula neck was measured in the axial view

All measurements were taken *via* the PACS system (Picture Archiving and Communication System). Software from GE Healthcare called Centricity™ Universal Viewer was used (RA1000, edition 2019, Buckinghamshire, Great Britain). The osteoarthritis score of each shoulder joint was classified according to Kellgren/Lawrence (KL^20^), to group patients in SJH and SJD. All glenoidal morphology parameters were measured after established methods. The glenoid distance in coronal (GDc) and axial view (GDa), the glenoid inclination (GI), the glenoid version (GV) as well as scapula neck length (SNL) were determined [21, 28, 29]. To determine GD, the length of the maximum extension of the glenoid articular surface was measured in both the coronal (GDc) and axial (GDa) dimensions. In order to calculate the GI, a tangent was first applied to the cranial and caudal boundaries of the glenoid in the coronal plane. A linear line was then placed through the superior border of the scapula. Of the angles formed at the intersection of the two lines, the inferior medial angle was used for further calculation. From this angle, 90° were subtracted, resulting in GI. The same principle was used to determine the tangent and corresponding straight line in the axial sectional plane for the measurement of GV. For the further calculation the ventral lateral angle at the crossing point was used. From this angle, 90° were subtracted, resulting in GV. To determine SNL, the widest extent from the joint surface side to the cortical end of the medial scapula neck was measured in the axial view. All measurements were carried out in a standardized manner on the best-represented layers with the largest measuring surface for each parameter. Figure 2 shows the principle of the measurement methodology. All radiographic parameters in this MRI study were manually measured separately in a standardized manner by the same observer (LEB) under supervision of an experienced senior radiologist (ASAH). Intraobserver reliability of the measurements of all parameters was assessed for a subset of 50 subjects by blinded re-evaluation at 2 weeks after the first measurement and using the same technique. Interobserver reliability was assessed by two observers (LEB and MPM) independently for 50 subjects.

### Statistics

For side-, age- and gender-specific analyses of GDc, GDa, GI, GV and SNL a Mann-Whitney U test was used. Likewise, Mann Whitney U test was implemented for comparison of SJH and SJD for all assessed parameters. The side-specific analysis included only one shoulder joint of the examined patients. No patient was examined bilaterally.

In order to detect possible interrelationships between the different glenoidal morphology parameters and patient age, a Spearman correlation was set. Intra- and interobserver reliabilities were evaluated using intraclass correlation coefficients (ICC). Overall, mean ± standard deviation is stated. Statistical analysis was performed with GraphPad Prism 9.00 (GraphPad Software, San Diego, USA), SPSS Statistics software version 27.0 (IBM SPSS Inc., Chicago, IL, USA) and Microsoft Excel (Microsoft Office 2016, Redmond, USA). Significant differences are marked with asterisks (***p < 0.001, **p < 0.01, *p < 0.05).

## Results

### Characteristics of the study population

In this study 235 males (56.2%) and 183 females (43.8%) were analysed. 228 right (54.6%) and 190 left (45.4%) shoulders were included in the final analysis. The mean age of the study population was 50.6 years (± 16.3 [age range: 20–89 years]).

### Analysis of intra-observer and inter-observer reliability

**Fig. 3:**
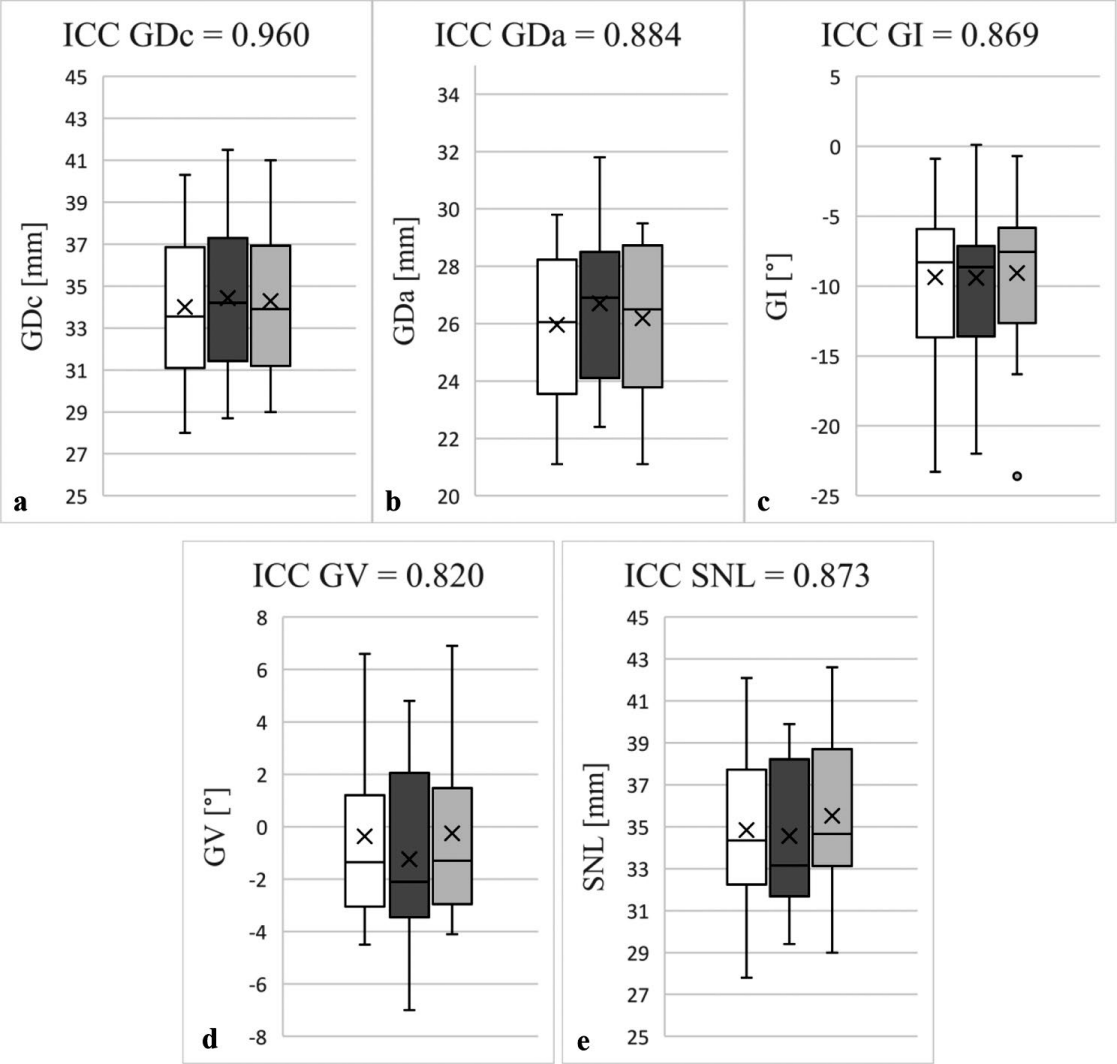
Determination of intraclass/interclass correlation coefficient (ICC) of glenoidal distance in coronal (GDc, **a**) and axial view (GDa, **b**), glenoidal inclination (GI, **c**), glenoidal version (GV, **d**) as well as scapula neck length (SNL, **e**): The white box plots represent the initial measurements by LEB, the dark grey ones the second recordings by LEB. The box plots marked in light grey reflect the measurements by MPM. The figures show the cumulative ICC between the initial and control measurements by LEB and the observations by MPM. Outliers were marked with points. Excellent measurement reliability (ICC > 0.9) was determined for GDc. GDa, GI, GV and SNL showed good investigation reliability (ICC between 0.75 and 0.9). The control measurements were carried out at intervals of two weeks by the same investigator again and another one in blinded fashion on the basis of 50 subjects

ICC for intra-observer reliability ranged from 0.82 to 0.96 and interobserver reliability from 0.80 to 0.95, indicating results between good and excellent reliability. Taking into account the initial measurements, the control measurements by the same examiner as well as the measurements by a second examiner, cumulative ICC values of 0.82 to 0.96 (GDc: 0.96; GDa: 0.88, GI: 0.87; GV: 0.82; SNL: 0.87) resulted. These results are summarised in Fig. 3.

### Analysis of glenoidal morphology parameters

Table 1 shows the descriptive analysis of glenoidal morphology in patients without radiological structural damage or radiological osteoarthritis (*n* = 418) including mean values, standard deviation, minimal and maximal measured values for GDc, GDa, GI, GV and SNL.

**Table 1:** Analysis of the mean value of the glenoid parameters with associated standard deviation (SD), minimum (min.) And maximum (max.)

(*n* = 418)		MW	SD	min.	max.
GDc	[mm]	33.4	± 3.6	24.4	48.0
GDa	[mm]	26.8	± 3.2	19.3	39.1
GI	[°]	10.5	± 6.4	-5.3	30.2
GV	[°]	-0.4	± 5.4	-14.4	19.0
SNL	[mm]	33.4	± 4.7	20.5	48.3

SD: Standard Deviation min.: minimale max.: maximal

#### Glenoidal distance in coronal view

In total (*n* = 418) a mean GDc of 33.4 mm (± 3.6 [24.4–48.0]) was found. The mean GDc in left shoulders (*n* = 190) was 33.1 mm (± 3.6) and 33.6 mm (± 3.7) on the right joint side (*n* = 228). For the younger patients (20–50 years, *n* = 194) a mean GDc of 32.7 mm (± 3.4) was detected, while the older patients (> 50 years, *n* = 224) showed a mean GDc of 34.0 mm (± 3.8). The analysis yielded a mean GDc of 30.0 mm (± 2.8) in female shoulders (*n* = 183) and of 35.2 mm (± 3.1) in male shoulders (*n* = 235).

#### Glenoidal distance in axial view

In total a mean GDa of 26.8 mm (± 3.2 [19.3–39.1]) was found. The mean GDa in left shoulders was 26.1 mm (± 3.2) and 27.4 mm (± 3.6) in right shoulders. For the younger age group, a mean GDa of 25.9 mm (± 3.2) was detected, while the older patients showed a mean GDa of 27.6 mm (± 3.5). The analysis yielded a mean GDa of 24.4 mm (± 2.5) in female shoulders and of 28.7 mm (± 3.0) in male shoulders.

#### Glenoid inclination

In total a mean GI of 10.5° (± 6.4 [-5.3-30.2]) was found. The mean GI in left shoulders was 11.0° (± 6.4) and in right shoulders 10.1° (± 5.9). For the younger age group, a mean GI of 10.7° (± 5.8) was detected, while the older patients showed a mean GI of 10.4° (± 6.5). The analysis yielded a mean GI of 11.1° (± 5.6) in female shoulders and of 10.0° (± 6.6) in male shoulders.

#### Glenoid version

In total a mean GV of -0.4° (± 5.4 [-14.4-19.0]) was found. The mean GV on the left shoulder joint was − 0.2° (± 5.4) and on the right joint side − 0.5° (± 5.3). For the younger age group, a mean GV of -0.7° (± 5.6) was detected. The older patients showed a mean GV of -0.1° (± 5.2). The analysis yielded a mean GV of 0.2° (± 5.1) in female shoulders and of -0.8° (± 5.5) in male shoulders.

#### Scapula neck length

In total a mean SNL of 33.4 mm (± 4.7 [20.5–48.3]) was found. The mean SNL in left shoulders was 33.4 mm (± 4.7) and also in right shoulders 33.4 mm (± 5.2). For the younger age group, a mean SNL of 33.0 mm (± 4.5) was detected, while the older patients showed a mean SNL of 33.8 mm (± 5.3). The analysis yielded a mean SNL of 31.6 mm (± 4.3) in female shoulders and of 34.8 mm (± 5.0) in male shoulders.

### Analysis of side specific differences for glenoidal morphology parameters

There was only a significant difference (*p* < 0.001) for GDa between left (26.1 mm [± 3.2]) and right (27.4 mm [± 3.6]) shoulder joints. No side-specific differences were found for GDc (*p* = 0.248), GI (*p* = 0.168), GV (*p* = 0.519) and SNL (*p* = 0.739). All results are summarised in Table 2.

**Table 2:** Analysis of side-specific differences between glenoid parameters

		total (*n* = 418)	left (*n* = 190)	right (*n* = 228)	*p*-value
GDc	[mm]	33.4 (± 3.6)	33.1 (± 3.6)	33.6 (± 3.7)	0.248^1^
GDa	[mm]	26.8 (± 3.2)	26.1 (± 3.2)	27.4 (± 3.6)	< 0.001***^1^
GI	[°]	10.5 (± 6.4)	11.0 (± 6.4)	10.1 (± 5.9)	0.168^1^
GV	[°]	-0.4 (± 5.4)	-0.2 (± 5.4)	-0.5 (± 5.3)	0.519^1^
SNL	[mm]	33.4 (± 4.7)	33.4 (± 4.7)	33.4 (± 5.2)	0.739^1^

^1^ Mann-Whitney U test

### Analysis of age specific differences and age depending correlation analysis for glenoidal morphology parameters

For GDc and GDa significant differences were found between the age groups (*p* < 0.001). The analysis showed a mean GDc of 32.7 mm (± 3.4) as well as GDa of 25.9 mm (± 3.2) in patients with an age of 20–50 years and a mean GDc of 34.0 mm (± 3.8) as well as GDa of 27.6 mm (± 3.5) in patients with an age over 50 years. There were no significant differences for GI (*p* = 0.376), GV (*p* = 0.097) and SNL (*p* = 0.131). These results are summarised in Table 3. Furthermore, an analysis of age-specific differences was performed separately for the left and right shoulder joints. Significant differences were observed for GDc (left: *p* = 0.008, right: *p* = 0.016) and GDa (left: *p* = 0.029; right: *p* < 0.001) as well. No significant differences in the body side separated age-specific analysis were detected for GI, GV and SNL. Tables 4 and 5 present these results in detail.

**Table 3:** Analysis of age-specific (age in years) differences between glenoid parameters

		total (*n* = 418)	20–50 y. (*n* = 194)	> 50 y. (*n* = 224)	*p*-value
GDc	[mm]	33.4 (± 3.6)	32.7 (± 3.4)	34.0 (± 3.8)	< 0.001***^1^
GDa	[mm]	26.8 (± 3.2)	25.9 (± 3.2)	27.6 (± 3.5)	< 0.001***^1^
GI	[°]	10.5 (± 6.4)	10.7 (± 5.8)	10.4 (± 6.5)	0.376^1^
GV	[°]	-0.4 (± 5.4)	-0.7 (± 5.6)	-0.1 (± 5.2)	0.097^1^
SNL	[mm]	33.4 (± 4.7)	33.0 (± 4.5)	33.8 (± 5.3)	0.131^1^

^1^ Mann-Whitney U test

**Table 4:** Analysis of age-specific (age in years) differences between glenoid parameters of included left shoulder joints

		total (*n* = 190)	20–50 y. (*n* = 79)	> 50 y. (*n* = 111)	*p*-value
GDc	[mm]	33.1 (± 3.6)	32.3 (± 3.5)	33.6 (± 3.6)	0.008**^1^
GDa	[mm]	26.1 (± 3.2)	25.5 (± 3.2)	26.5 (± 3.1)	0.029*^1^
GI	[°]	11.0 (± 6.4)	11.5 (± 5.9)	10.6 (± 6.8)	0.137^1^
GV	[°]	-0.2 (± 5.4)	-0.2 (± 5.9)	-0.2 (± 5.2)	0.864^1^
SNL	[mm]	33.4 (± 4.7)	33.2 (± 4.4)	33.5 (± 4.9)	0.569^1^

^1^ Mann-Whitney U test

**Table 5:** Analysis of age-specific (age in years) differences between glenoid parameters of included right shoulder joints

		total (*n* = 228)	20–50 y. (*n* = 106)	> 50 y. (*n* = 122)	*p*-value
GDc	[mm]	33.6 (± 3.7)	33.0 (± 3.4)	34.2 (± 3.9)	0.016*^1^
GDa	[mm]	27.4 (± 3.6)	26.2 (± 3.3)	28.4 (± 3.6)	< 0.001***^1^
GI	[°]	10.1 (± 5.9)	10.1 (± 5.7)	10.1 (± 6.2)	0.949^1^
GV	[°]	-0.5 (± 5.3)	-0.9 (± 5.2)	-0.1 (± 5.4)	0.148^1^
SNL	[mm]	33.4 (± 5.2)	32.8 (± 4.6)	33.9 (± 5.7)	0.183^1^

^1^ Mann-Whitney U test

**Fig. 4:**
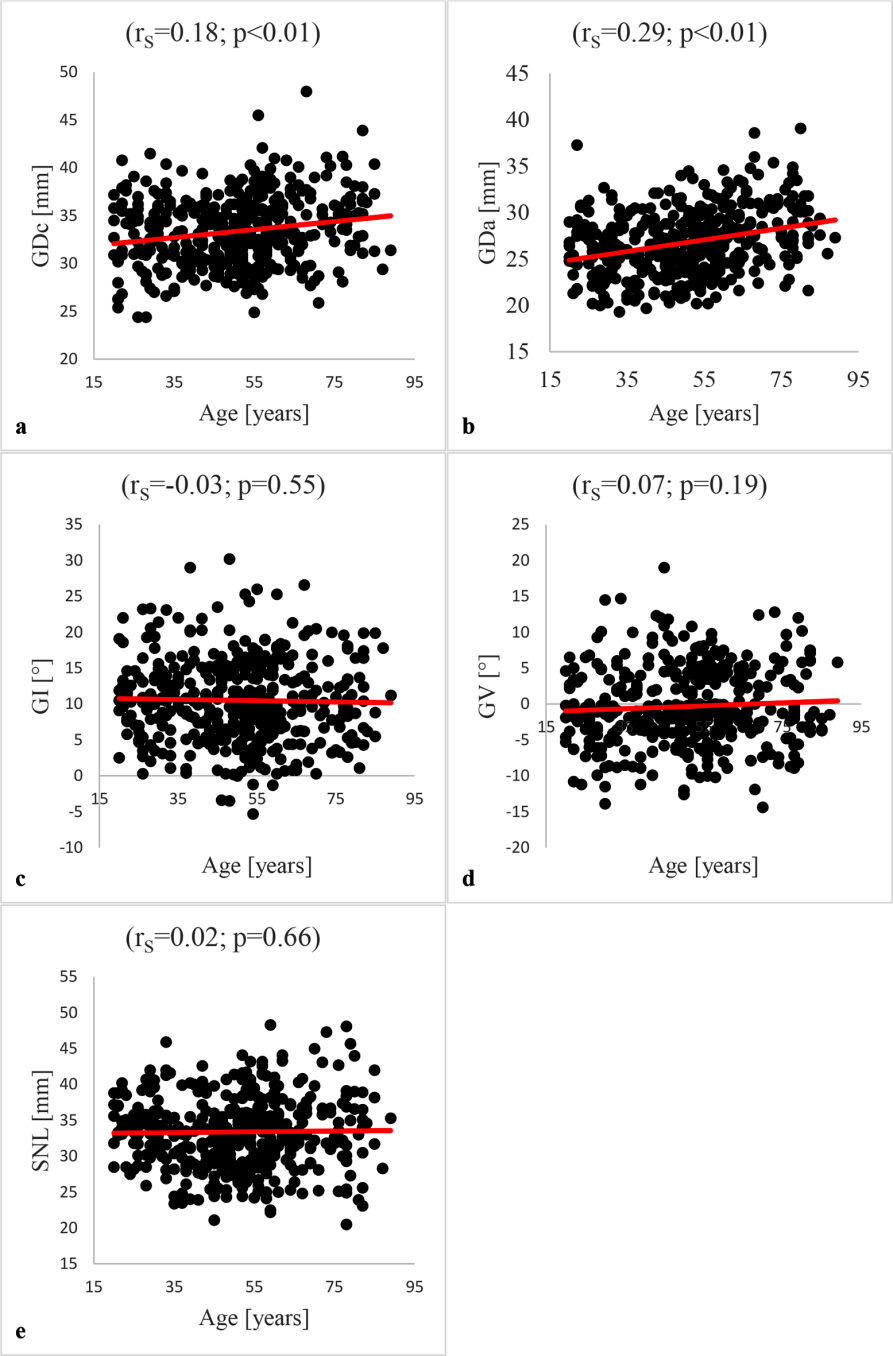
Correlation analysis between patients age and GDc, GDa, GI, GV and SNL: The analysis based on Spearman correlation. The analysis detected a significant correlation of GDc (r_S_: 0.18; *p* < 0.01;) (**a**) and GDa (r_S_: 0.29; *p* < 0.01;) (**b**) with patient age. There was no significant correlation between patient age and GI (r_S_=-0.03; *p* = 0.55) (**c**), GV (r_S_=0.07; *p* = 0.19) (**d**) respectively SNL (r_S_=0.02; *p* = 0.66) (**e**)

The correlation analysis showed weak significant (*p* < 0.001) correlations between age and GDc (r_S_=0.181) respectively GDa (r_S_=0.290). There were no significant correlations between age and GI (*p* = 0.549), GV (*p* = 0.189) as well as SNL (*p* = 0.661). The results of the correlation analysis are summarised in Fig. 4.

### Analysis of gender specific differences for glenoidal morphology parameters

For all parameters with the exception of GV (*p* = 0.079) significant gender-associated differences were detected. The mean GDc in female patients was 30.0 mm (± 2.8) and 35.2 mm (± 3,1) in male patients (*p* < 0.001). Likewise, a significant (*p* < 0.001) difference in GDa was found between male shoulder joints (28.7 mm [± 3.0]) and females (24.4 mm [± 2.5]). GI showed a significant (*p* = 0.021) different mean in the women group (11.1° [± 5.6]) in comparison to the men group (10.0° [± 6.6.]). Furthermore, SNL was significant (*p* < 0.001) different between male shoulder joints (34.8 mm [± 5.0]) and the female ones (31.6 mm [± 4.3]). These results are summarised in Table 6. Moreover, an analysis of gender-specific differences was performed separately for the left and right shoulder joints. Significant differences were found for GDc (left and right each: *p* < 0.001), GDa (left and right each: *p* < 0.001), GI (left: *p* = 0.049, right: *p* = 0.037) and SNL (left and right each: *p* < 0.001) as well. No significant differences in the body side separated gender-specific analysis were detected for GV. Tables 7 and 8 present these results in detail.

**Table 6:** Analysis of gender-specific differences between glenoid parameters

		total (*n* = 418)	females (*n* = 183)	males (*n* = 235)	*p*-value
GDc	[mm]	33.4 (± 3.6)	30.0 (± 2.8)	35.2 (± 3.1)	< 0.001***^1^
GDa	[mm]	26.8 (± 3.2)	24.4 (± 2.5)	28.7 (± 3.0)	< 0.001***^1^
GI	[°]	10.5 (± 6.4)	11.1 (± 5.6)	10.0 (± 6.6)	0.021*^1^
GV	[°]	-0.4 (± 5.4)	0.2 (± 5.1)	-0.8 (± 5.5)	0.079^1^
SNL	[mm]	33.4 (± 4.7)	31.6 (± 4.3)	34.8 (± 5.0)	< 0.001***^1^

^1^ Mann-Whitney U test

**Table 7:** Analysis of gender-specific differences between glenoid parameters of included left shoulder joints

		total (*n* = 190)	females (*n* = 93)	males (*n* = 97)	*p*-value
GDc	[mm]	33.1 (± 3.6)	30.8 (± 2.7)	35.2 (± 3.0)	< 0.001***^1^
GDa	[mm]	26.1 (± 3.2)	23.9 (± 2.2)	28.1 (± 2.6)	< 0.001***^1^
GI	[°]	11.0 (± 6.4)	11.5 (± 4.9)	10.5 (± 7.6)	0.049*^1^
GV	[°]	-0.2 (± 5.4)	0.3 (± 5.1)	-0.7 (± 5.8)	0.186^1^
SNL	[mm]	33.4 (± 4.7)	31.7 (± 4.0)	35.0 (± 4.7)	< 0.001***^1^

^1^ Mann-Whitney U test

**Table 8:** Analysis of gender-specific differences between glenoid parameters of included right shoulder joints

		total (*n* = 228)	females (*n* = 90)	males (*n* = 138)	*p*-value
GDc	[mm]	33.6 (± 3.7)	31.1 (± 2.9)	35.3 (± 3.2)	< 0.001***^1^
GDa	[mm]	27.4 (± 3.6)	24.9 (± 2.8)	29.1 (± 3.1)	< 0.001***^1^
GI	[°]	10.1 (± 5.9)	10.8 (± 6.2)	9.6 (± 5.7)	0.037*^1^
GV	[°]	-0.5 (± 5.3)	0.1 (± 5.1)	-0.8 (± 5.4)	0.250^1^
SNL	[mm]	33.4 (± 5.2)	31.4 (± 4.6)	34.6 (± 5.3)	< 0.001***^1^

^1^ Mann-Whitney U test

### Analysis between glenoidal morphology parameters and grade of osteoarthritis

403 (96,4%) patients were graded as KL 0–2 (SJH) and 15 (3.6%) patients were graded as KL 3–4 (SJD). The SJH group included 186 left und 217 right shoulder joints, 183 patients with an age of 20–50 years and 220 patients with an age over 50 years. Furthermore, 175 female and 228 male shoulders were classified as KL 0–2. In the SJD group were 4 left and 11 right shoulder joints. 2 Patients had an age between 20 and 50 years, while 13 patients were older than 50 years. 8 women and 7 men were included in the SJD group. There was a significant difference (*p* = 0.027) between the mean GDc of the SJH (33.3 mm [± 3.6]) compared to the mean GDc of the SJD (36.1 mm [± 4.8]). Likewise, GDa showed a significant difference (*p* = 0.017) between the groups (SJH: 26,7 mm [± 3.4]; SJD: 29.3 mm [± 4.0]). The other measured parameters showed no significant differences in comparison of both groups (GI: *p* = 0.676; GV: *p* = 0.341; SNL: *p* = 0.576). The results are summarised in Table 9. More specifically, separate page, age and gender analyses were carried out in the SJH and SJD groups. The side-specific analysis showed significant differences for GDc (*p* = 0.048) and GDa (*p* < 0.001) in patients classified as KL 0–2. In the SJD group no significant differences were detected. Likewise, GDc (*p* = 0.002) and GDa (*p* < 0.001) differed significantly between patients with an age between 20 and 50 and over 50 years in SJH, but not in SJD. With the exception of GV (*p* = 0.051), significant differences were found between female and male shoulder joint in the SJH group (GDc: *p* < 0.001, GDa: <0.001, GI: *p* = 0.024 and SNL: *p* < 0.001). The analysis of SJD revealed only a gender-specific difference for GDc (*p* = 0.026). Tables 10, 11, 12, 13, 14 and 15 present these results in detail.

**Table 9:** Analysis of osteoarthritis-specific differences between glenoid parameters

		total (*n* = 418)	KL 0–2 (*n* = 403)	KL 3–4 (*n* = 15)	*p*-value
GDc	[mm]	33.4 (± 3.6)	33.3 (± 3.6)	36.1 (± 4.8)	0.027*^1^
GDa	[mm]	26.8 (± 3.2)	26.7 (± 3.4)	29.3 (± 4.0)	0.017*^1^
GI	[°]	10.5 (± 6.4)	10.5 (± 6.2)	10.8 (± 4.5)	0.676^1^
GV	[°]	-0.4 (± 5.4)	-0.3 (± 5.3)	-1.8 (± 6.0)	0.341^1^
SNL	[mm]	33.4 (± 4.7)	33.4 (± 5.0)	32.7 (± 5.5)	0.576^1^

^1^ Mann-Whitney U test

**Table 10:** Analysis of side-specific differences between glenoid parameters of included shoulder joints with a Kellgren/Lawrence score of 0–2

		total (*n* = 403)	left (*n* = 186)	right (*n* = 217)	*p*-value
GDc	[mm]	33.3 (± 3.6)	33.0 (± 3.6)	33.6 (± 3.6)	0.048*^1^
GDa	[mm]	26.7 (± 3.4)	26.0 (± 3.1)	27.3 (± 3.6)	< 0.001***^1^
GI	[°]	10.5 (± 6.2)	10.9 (± 6.5)	10.1 (± 6.0)	0.230^1^
GV	[°]	-0.3 (± 5.3)	-0.1 (± 5.4)	-0.4 (± 5.3)	0.485^1^
SNL	[mm]	33.4 (± 5.0)	33.4 (± 4.7)	33.4 (± 5.2)	0.920^1^

^1^ Mann-Whitney U test

**Table 11:** Analysis of side-specific differences between glenoid parameters of included shoulder joints with a Kellgren/Lawrence score of 3–4

		total (*n* = 15)	left (*n* = 4)	right (*n* = 11)	*p*-value
GDc	[mm]	36.1 (± 4.8)	36.9 (± 2.8)	35.8 (± 5.6)	0.508^1^
GDa	[mm]	29.3 (± 4.0)	30.0 (± 5.3)	29.0 (± 4.0)	0.973^1^
GI	[°]	10.8 (± 4.5)	13.4 (± 3.2)	9.8 (± 4.9)	0.188^1^
GV	[°]	-1.8 (± 6.0)	-3.5 (± 6.5)	-1.2 (± 6.4)	0.681^1^
SNL	[mm]	32.7 (± 5.5)	34.4 (± 1.5)	32.1 (± 6.5)	0.280^1^

^1^ Mann-Whitney U test

**Table 12:** Analysis of age-specific (age in years) differences between glenoid parameters of included shoulder joints with a Kellgren/Lawrence score of 0–2

		total (*n* = 403)	20–50 y. (*n* = 183)	> 50 y. (*n* = 220)	*p*-value
GDc	[mm]	33.3 (± 3.6)	32.6 (± 3.4)	33.8 (± 3.6)	0.002**^1^
GDa	[mm]	26.7 (± 3.4)	25.8 (± 3.1)	27.4 (± 3.5)	< 0.001***^1^
GI	[°]	10.5 (± 6.2)	10.7 (± 5.8)	10.3 (± 6.6)	0.411^1^
GV	[°]	-0.3 (± 5.3)	-0.6 (± 5.5)	-0.1 (± 5.2)	0.166^1^
SNL	[mm]	33.4 (± 5.0)	33.0 (± 4.5)	33.8 (± 5.3)	0.136^1^

^1^ Mann-Whitney U test

**Table 13:** Analysis of age-specific (age in years) differences between glenoid parameters of included shoulder joints with a Kellgren/Lawrence score of 3–4

		total (*n* = 15)	20–50 y. (*n* = 2)	> 50 y. (*n* = 13)	*p*-value
GDc	[mm]	36.1 (± 4.8)	39.2 (± 2.6)	35.6 (± 5.1)	0.219^1^
GDa	[mm]	29.3 (± 4.0)	34.0 (± 4.7)	28.6 (± 3.8)	0.219^1^
GI	[°]	10.8 (± 4.5)	14.6 (± 0.0)	10.2 (± 4.8)	0.295^1^
GV	[°]	-1.8 (± 6.0)	-1.6 (± 7.0)	-1.9 (± 6.4)	0.952^1^
SNL	[mm]	32.7 (± 5.5)	31.8 (± 3.1)	32.9 (± 6.0)	0.800^1^

^1^ Mann-Whitney U test

**Table 14:** Analysis of gender-specific differences between glenoid parameters of included shoulder joints with a Kellgren/Lawrence score of 0–2

		total (*n* = 403)	females (*n* = 175)	males (*n* = 228)	*p*-value
GDc	[mm]	33.3 (± 3.6)	30.8 (± 2.7)	35.1 (± 3.0)	< 0.001***^1^
GDa	[mm]	26.7 (± 3.4)	24.3 (± 2.4)	28.6 (± 2.9)	< 0.001***^1^
GI	[°]	10.5 (± 6.2)	11.1 (± 5.6)	10.0 (± 6.6)	0.024*^1^
GV	[°]	-0.3 (± 5.3)	0.3 (± 5.1)	-0.8 (± 5.5)	0.051^1^
SNL	[mm]	33.4 (± 5.0)	31.6 (± 4.2)	34.8 (± 5.1)	< 0.001***^1^

^1^ Mann-Whitney U test

**Table 15:** Analysis of gender-specific differences between glenoid parameters of included shoulder joints with a Kellgren/Lawrence score of 3–4

		total (*n* = 15)	females (*n* = 8)	males (*n* = 7)	*p*-value
GDc	[mm]	36.1 (± 4.8)	33.4 (± 3.2)	39.2 (± 4.9)	0.026*^1^
GDa	[mm]	29.3 (± 4.0)	27.3 (± 3.7)	31.6 (± 3.5)	0.056^1^
GI	[°]	10.8 (± 4.5)	11.3 (± 4.4)	10.2 (± 5.3)	0.677^1^
GV	[°]	-1.8 (± 6.0)	-2.6 (± 4.2)	-0.9 (± 8.3)	0.635^1^
SNL	[mm]	32.7 (± 5.5)	31.9 (± 7.1)	33.7 (± 3.7)	0.336^1^

^1^ Mann-Whitney U test

## Discussion

Shoulder instability is a clinical problem in joint preserving and replacing surgery [4–8, 15, 16]. The morphological reconstruction of the glenoid in the event of a traumatic injury as well as the positioning of the glenoid component in case of anatomic or reverse total arthroplasty are crucial for joint stability [13, 15, 16]. In order to achieve optimal postoperative results, physiological reference values of the morphology of the glenoid are required, which are insufficiently described in the current literature [21–23]. Therefore, it was aimed to define physiological radiological reference values for the morphology of the glenoid. For this task, in the present study, MRI scans of 418 shoulder joints were examined in a low-level osteoarthritis collective to establish such reference values in order to optimize the diagnostics and therapy of shoulder joint instability in the native and arthroplasty joint.

Analysis of side-specific differences showed only a significant result for GDa, which was higher on the right joint side compared to the left side. A possible explanation for this difference is that 90% of the population is right-handed and therefore uses the right upper limb more dominantly [30, 31]. However, due to the retrospective nature of the present study, this assumption can only be made hypothetically. A comparative study by Karademir et al. found contrasting results. In a retrospective approach, the authors examined the glenoid diameters of 102 shoulder joints using computerized topographic (CT) images [21]. No significant differences between left- and right-handers in the side-specific analysis were detected. However, the publication reveals no differentiation between the coronal and axial glenoid diameters, which could be a reason for the different study results, as the present study only found significant side-specific differences for GDa, but not for GDc. However, the side-dependent analysis of patients with a KL score of 0–2 showed a significant difference for GDc, with larger values recorded on the right shoulder joints. In SJD patients no significant differences were revealed for GDc and GDa, which could be caused by the small group size (*n* = 15). Finally concluding, it needs to be recognized that the results of the present study do not present a clear pattern either. In order to finally clarify the question of an existing side dependency of GDc and GDa, additional studies are necessary that specifically examine differentiated GDc and GDa between left- and right-handers, ideally in a prospective design. Assuming that the present results could only detect a significant lateral difference for GDa and that this is very small (left: 26.1 mm [± 3.2]; right 27.4 mm [± 3.6]), it should be considered as a clinical consequence to use the healthy opposite side as a reference for the reconstruction of the physiological glenoid morphology in the preoperative diagnostics of shoulder joint instability in the native joint or the planning of a shoulder joint replacement. This concept is clinically well established for hip joint arthroplasty [32, 33].

Age-dependent significant differences were found for GDc and GDa. In addition, a weak correlation between GDc and GDa with patient age was detected. To exclude a potential bias due to the side of the body, the analyses were also carried out separately for the left and right shoulder joints between the two age groups. Here, age-specific differences were also found between GDc and GDa, so that a bias due to the side of the body can be excluded with high probability. Taking into account the osteoarthritis-dependent analysis, in which significantly higher measured values for GDc and GDa were found in the group with a higher grade of osteoarthritis (KL 3–4), the most plausible reason is degenerative remodelling of the morphological of osseous glenoid structure. The most likely cause for the increase in GDc and GDa with higher patient age appears to be the formation of osteophytic marginal attachments. These already occur at a KL grade of 2 and could therefore also be present in the SJH group. This hypothesis could be confirmed by the results of the KL score-based analysis, since significantly greater values were obtained in patients older than 50 years with a KL score of 0–2 in the age-specific analysis of GDc and GDa. Although no significant differences were found in patients with a KL 3–4, this circumstance could be due to the small group size (*n* = 15), as explained above. An increase in the size of the glenoid diameters in advanced osteoarthritis of the shoulder joint has already been described by Mullaji et al. [34]. In a retrospective analysis of 64 CT examinations, an increase in the anteroposterior diameter of 5 to 8 mm was found. Pfahler et al. found age-specific remodelling in the area of the glenoidal labral complex in a cadaver study of 32 shoulder joints without signs of advanced osteoarthritis [35]. The authors attributed this to increased mechanical stress in this area. The enhanced mechanical stress exposure could also be a trigger for remodelling of the adjoining bony structures of the glenoid and thereby explain the age-dependent increase in GDc and GDa demonstrated in the presented study. An understanding of the age- and osteoarthritis-related changes in GDc and GDa is of major importance for the preoperative planning of total shoulder arthroplasty in order to avoid malpositioning of the glenoid component and the subsequent instabilities/dislocations [16–18]. The original physiological joint morphology should be reconstructed in order to achieve exact positioning of the glenoid component [15, 16, 36, 37]. As a conclusion, all bony overgrowths need to be resected intraoperatively before positioning the glenoid component. The results of the younger patient group presented in this study can serve as a physiological reference.

The analysis revealed significantly higher measurement results for GDc, GDa and SNL in the male patients compared to the female patients. A gender-dependency of glenoidal morphology parameters has already been reported in other studies. For example, Piponov et al. demonstrated a greater glenoid height and diameter in coronal view of male patients, by retrospectively analysing 108 shoulder CT scans [38]. Likewise, higher measurement values for glenoid height and width were reported by Mathews et al., who examined 18 body donors to detect potential sex-specific differences [39]. One logical explanation for these results is the greater average body height of men in comparison to women [40, 41]. In contrast, a significantly higher GI was found in the female compared to the male shoulder joints. In general, there are only a few studies in the current literature that compare GI between the genders. However, in a cadaver study of 344 scapulae, Churchill et al. found no sex-specific differences in GI [42]. Further reference studies are needed to finally clarify the question whether a gender-dependency of GI exist. GI is important for shoulder stability resurfaced joints. A superior inclination after reversed shoulder arthroplasty is a risk factor for joint instability [43]. Based on the results of the present study, a possible gender dependency of GI should therefore be taken into account in the diagnostics of shoulder joint instability after joint replacement therapy. To exclude a potential bias due to the side of the body, the analyses of gender-specificity were also carried out separately for the left and right shoulder. The significances were congruent with the overall analysis. It is not assumed that there is a bias. Furthermore, there not seemed to be a bias due to osteoarthritis level of shoulder joint, while the significant differences were congruent in the separated analysis of patients with a KL score of 0–2, too. Even though the analysis of patients with a KL score of 3–4 only yielded significant differences for GDc, the small group size (*n* = 15) is considered to be the most likely cause of the lack of significant differences in the other parameters, as already mentioned. No significant differences were found for the GV in any of the analyses. However, a slight retroversion/neutral position of the glenoid was found on average in the overall collective. In the current literature, there is a wide variation in the reference values given for the GV, ranging from retroversion (-16°) to anteversion (+ 21°) [44–47]. Based on the results of the present study, a neutral position for the GV is recommended both for the surgical treatment of instability in case of shoulder joint dysplasia in young patients and for the positioning of the glenoid component during joint replacement.

In summary, the presented results should be used a physiological reference of glenoid morphology of the adult shoulder in daily clinical practice and should optimize the diagnostics and therapy of shoulder instability.

A limitation of the study is that there was no comparison of the MRI scans with the related x-ray imaging, because many of the included patients were only examined via MRI, but not via x-ray. A comparison between the different modalities seems useful. The present study could thereby have made an even better comparison with the results of other authors. In addition, a comparison between measurements from MRI images and CT scans would be desirable. Compared to MRI, the CT in general is more precise when it comes to assessing bone structures. Another important point that needs to be mentioned as a limitation of the study results is that the analysis of the influencing factors of side, age and gender on the morphological parameters ware only carried out one-dimensionally. Combined dependencies of, as an example, age and gender remain unconsidered.

In summary, the MRIs of 418 patients were analysed to define physiological reference values for glenoid morphology in a collective of predominantly healthy shoulder joints. The Kellgren/Lawrence Score was used to verify presence of a low-grade osteoarthritic population. Mean values for GDc, GDa, GI, GV and SNL depending on joint side, patient age, gender and degenerative changes were presented. Age- and gender-specific significant differences were detected. With the exception of GDa, no side-specific differences were observed. The results suggest that preoperative planning of reconstruction of the physiological glenoid morphology on the healthy opposite side should be considered in everyday clinical practice. The glenoidal distance parameters (GDc and GDa) appear to increase as part of the ageing process due to degenerative changes. GDc, GDa and SNL were significantly larger in the examined men than in the women, which can be explained by the greater average height of men. In contrast, the female shoulders showed a significantly higher mean GI. Interestingly, the analysis of the GV in the entire collective revealed a slight retroversion of the glenoid on average. Even considering that further studies are needed to verify the results, they should be recognized in the future as a physiological reference for the morphology of the adult glenoid in order to improve the diagnostics and therapy of shoulder joint instability in the native and resurfaced joint.

## Data Availability

All data generated or analysed during this study are included in this published article.

## Abbreviations

CT: Computed Tomography
FOV: Field-Of-View
GDa: Glenoidal Distance in axial view
GDc: Glenoidal Distance in coronal view
GI: Glenoid Inclination
GV: Glenoid Version
ICC: Intraclass Correlation Coefficients
KL: Kellgren/Lawrence
MRI: Magnetic Resonance Imaging
OA: Osteoarthritis
PD: Proton Density-weighted
SJD: Degenerative Shoulder joint
SJH: Healthy Shoulder Joint
SNL: Scapula Neck Length
SE: Spin Echo
